# Role of Peroxisome Proliferator-Activated Receptor **β**/**δ** and B-Cell Lymphoma-6 in Regulation of Genes Involved in Metastasis and Migration in Pancreatic Cancer Cells

**DOI:** 10.1155/2013/121956

**Published:** 2013-05-02

**Authors:** Jeffrey D. Coleman, Jerry T. Thompson, Russell W. Smith, Bogdan Prokopczyk, John P. Vanden Heuvel

**Affiliations:** ^1^Department of Veterinary and Biomedical Sciences and Center for Molecular Toxicology and Carcinogenesis, Penn State University, 325 Life Sciences Building, University Park, PA 16802, USA; ^2^Department of Pharmacology, Penn State University, Hershey, PA 17033, USA; ^3^Penn State Cancer Institute, Hershey, PA 17033, USA; ^4^Indigo Biosciences Inc., State College, PA 16801, USA

## Abstract

*PPARβ*/*δ* is a ligand-activated transcription factor that regulates various cellular functions via induction of target genes directly or in concert with its associated transcriptional repressor, *BCL-6*. Matrix remodeling proteinases are frequently over-expressed in pancreatic cancer and are involved with metastasis. The present study tested the hypothesis that *PPARβ*/*δ* is expressed in human pancreatic cancer cells and that its activation could regulate *MMP-9*, decreasing cancer cells ability to transverse the basement membrane. In human pancreatic cancer tissue there was significantly higher expression of *MMP-9* and *PPARβ*/*δ*, and lower levels of *BCL-6* mRNA. *PPARβ*/*δ* activation reduced the TNF**α**-induced expression of various genes implicated in metastasis and reduced the invasion through a basement membrane in cell culture models. Through the use of short hairpin RNA inhibitors of *PPARβ*/*δ*, *BCL-6*, and *MMP-9*, it was evident that *PPARβ*/*δ* was responsible for the ligand-dependent effects whereas *BCL-6* dissociation upon GW501516 treatment was ultimately responsible for decreasing *MMP-9* expression and hence invasion activity. These results suggest that *PPARβ*/*δ* plays a role in regulating pancreatic cancer cell invasion through regulation of genes via ligand-dependent release of *BCL-6* and that activation of the receptor may provide an alternative therapeutic method for controlling migration and metastasis.

## 1. Introduction

Pancreatic cancer is the fourth leading cause of cancer-related deaths of men and women in the United States. The American Cancer Society estimates for 2009 predicted approximately 42,470 new cases of pancreatic cancer and that 35,240 of those cases would result in death. Lack of identifiable symptoms or biomarkers combined with a 4% five-year survival rate makes pancreatic cancer one of the deadliest malignancies [1]. Although pancreatic cancer is difficult to detect in its early stages, several known risk factors exist, with smoking being the most well-documented etiologic agent [2]. Several other risk factors include age, diets high in fat [3], excessive alcohol consumption [4], diabetes mellitus [5], and chronic pancreatitis [6]. Common chemotherapeutic treatments have had little success in improving survival rates or restraining the highly metastatic malignancies [7] with the median survival rate of less than six months and surgical resection as the only effective treatment [8]. Prevention strategies and alternative treatments for pancreatic cancer are sorely needed.

Peroxisome proliferator-activated receptor-*β*/*δ* (*PPARβ*/*δ*) is a member of the nuclear receptor superfamily of ligand-activated transcription factors. The *PPAR*s consist of three isoforms: *PPARα* (*NR1C1*), *PPARβ*/*δ* (*NR1C2*; *NUC1*; *FAAR* fatty acid-activated receptor), and *PPARγ* (*NR1C3*). The *PPAR*s effect gene transcription in response to various stimuli, such as fatty acids and their metabolites, xenobiotics and isoform-specific drugs, through a heterodimerization with retinoid *X* receptors (*RXR*s) and subsequent recognition and binding to peroxisome proliferator-responsive elements (PPREs) within the promoter regions of target genes [9, 10]. *PPARβ*/*δ*, unlike *PPARα* or *PPARγ* which have distinct tissue expression patterns and synthetic ligands, is ubiquitously expressed, often at higher levels than the other isoforms. This receptor regulates fatty acid oxidation and lipid homeostasis [11], cell proliferation and differentiation [12], cell survival [13], and the inflammatory response [14]. The latter response may be via its association with the transcriptional repressor *BCL-6*, which is released upon activation of *PPARβ*/*δ* [15]. In the pancreas, *PPARβ*/*δ* is expressed in islet cells to a greater extent than either *PPARα* or *PPARγ* and in beta cells where it regulates the inflammatory response [16]. Expression profiling analyses in the mouse demonstrated high *PPARβ*/*δ* expression in the cytoplasm of delta cells of the islet of Langerhans, suggesting a potential role for the receptor in the regulation of glucose metabolism [17]. Pancreatic ductal adenocarcinomas are by far the most common of pancreatic malignancies [18], and the role(s) of *PPARβ*/*δ* in pancreatic ductal cells is poorly understood. 

The matrix metalloproteinases are a family of zinc-dependent proteolytic enzymes that degrade extracellular matrix (ECM) proteins and are well-known regulators of pancreatic cancer cell metastasis and invasion [19, 20]. Matrix metalloproteinase-9 (*MMP-9*, also known as gelatinase B) in particular is highly expressed in both clinical and experimental models of pancreatic cancer [21]. Furthermore, pancreatic cancer cells display extremely high basal *MMP-9* expression, which is further inducible by phorbol 12-myristate 13-acetate (PMA) [22]. Recently, several studies have linked *PPARβ*/*δ* to *MMP-9*; in *PPARβ*/*δ* null macrophages, basal *MMP-9* expression is reduced [15], and in vascular smooth muscle cells (VSMCs) *PPARβ*/*δ* activation suppressed the expression of both *MMP-2* and *MMP-9*, with further inhibition on VSMC migration and proliferation [23]. 

The role(s) of *PPAR*s, particularly *PPARβ*/*δ*, in tumorigenesis and cancer cell invasion remains controversial. For example, inhibition of *PPARγ* suppressed pancreatic cancer cell motility in Capan-1 and Panc-1 cells [24], while its activation in AsPC-1 cells by the specific ligand rosiglitazone increased levels of the tumor suppressor *PTEN* and decreased levels of phosphorylated Akt [25] and induced caspase-mediated apoptosis in Miapaca-2 cells [26]. *PPARβ*/*δ* is an APC-regulated target of nonstreroidal anti-inflammatory drugs (NSAIDs), suggesting that NSAIDs inhibit tumorigenesis via *PPARβ*/*δ* inhibition [27], and genetic disruption of *PPARβ*/*δ* contributes to the growth-inhibitory effects of APC [28]. Opposing evidence exists suggesting that *PPARβ*/*δ* activation increases [29–31] and decreases cell proliferation [32, 33] in various cell types. Previous evidence, however, establishes a clear link between *PPARβ*/*δ, BCL-6,* and *MMP-9*, and we sought to elucidate the role(s) of *PPARβ*/*δ* activation on potential target genes involved in pancreatic cancer invasion and metastasis. The *PPARβ*/*δ-*specific activator GW501516 and shRNAs to decrease expression of *PPARβ*/*δ, BCL-6,* and *MMP-9* were used in two human pancreatic cancer cell lines, Miapaca-2 (*COX-2* negative) and BxPc-3 (*COX-2* positive). The experiments show that ligand-dependent activation of *PPARβ*/*δ* causes a *BCL6*-dependent repression of *MMP-9* and other genes involved in cancer metastasis and decreases indices of cell migration, suggesting that *PPARβ*/*δ* agonists may be a beneficial tool in the prevention and treatment of pancreatic cancer.

## 2. Materials and Methods

### 2.1. Cells and Reagents

Human pancreatic cancer cells, Miapaca-2 (*COX-2* negative, CRL-1420) and BxPc-3 (*COX-2* positive, CRL-1687), were purchased from the ATCC (Manassas, VA) and cultured in high-glucose DMEM containing 10% FBS and 1% penicillin/streptomycin in a humidified atmosphere at 37°C containing 5% CO_2_. Human embryonic kidney 293 cells were cultured in DMEM containing 10% FBS and 1% penicillin/streptomycin. All media components and fetal bovine serum (FBS) were purchased from Gibco BRL/Life Technologies (Carlsbad, CA). Ciprofibrate (Cipro), purchased from Sigma Chemical Co. (St Louis, MO), was used as the positive control for *PPARα*. GW501516 (GW), purchased from Sigma Chemical Co., was used as the positive control for *PPARβ*/*δ*. Rosiglitazone (rosi), purchased from Cayman Chemicals (Ann Arbor, MI), was used as the positive control for *PPARγ*. Recombinant human *TNFα* and human *MMP-9* ELISAs were purchased from Invitrogen (Carlsbad, CA) and used according to the manufacturer's instructions. Human pancreatic cancer, chronic pancreatitis, and pancreas tissue samples were obtained from Dr. Gerhard Leder, (Abt. Allgemein-und Viszeralchirurgie, St. Josef Hospital—Klinikum der Ruhr, University of Bochum, Germany). MISSION shRNA bacterial glycerol stocks targeted against human *PPARβ*/*δ, Bcl6, MMP-9*, as well as the nontargeting vector, were purchased directly from Sigma-Aldrich. High Capacity cDNA Archive Kit and ABI7300 real-time PCR system were purchased from Applied Biosystems (Foster City, CA). The pPACKH1 packaging plasmids were kindly provided by Dr. Curtis J. Omiecinski (Penn State University). CytoSelect 96-well cell invasion assay (basement membrane, fluorometric format) was purchased from Cell Biolabs, Inc. (San Diego, CA) and used according to the manufacturer's instructions. 

### 2.2. Isolation of Total RNA and Real-Time Quantitative RT-PCR

Total RNA was isolated from Miapaca-2 and BxPc-3 cells using Tri-Reagent and the manufacturer's recommended protocol (Sigma). Human pancreatic tissue samples were briefly homogenized in 1 mL Tri-Reagent, and total RNA was isolated. One *μ*g of total RNA was reverse-transcribed using the High Capacity cDNA Archive Kit (Applied Biosystems, Foster City, CA). PCR primers for quantitative real-time RT-PCR were designed based on published sequences in GenBank and are shown in Table 1 in Supplementary Material available online at http://dx.doi.org/10.1155/2013/121956. The housekeeping gene *β-actin* was used to normalize all the tested genes. The data shown are representative of three independent experiments with triplicate samples.

### 2.3. Quantification of *MMP*-*9* Protein by ELISA

*MMP-9* protein levels were quantified using the human *MMP-9* ELISA according to the manufacturer's instructions (Invitrogen). Briefly, control Miapaca-2 cells or shRNA knockdown cells were plated in 6-well tissue culture plates and treated with 1 ng/mL *TNFα* with or without 500 nM GW501516 for 24 h. At the end of the incubation time, the media was removed and diluted 1 : 40 in standard diluent buffer. Diluted media samples and *MMP-9* standards were added to a 96-well microtiter plate containing human *MMP-9* antibody-coated wells and allowed to incubate at room temperature for 2 h. Following the incubation, the media was aspirated and each well washed 5 times with wash buffer. One hundred *μ*L Biotinylated anti-*MMP-9* (biotin conjugate) solution was added to each well, and the plate was incubated for 1 h at room temperature. Each well was washed a second time with wash buffer, and 100 *μ*L of streptavidin-HRP working solution was added and the plate was allowed to incubate at room temperature for 30 minutes. After a third wash, 100 *μ*L of stabilized chromogen was added to each well, and the plate was incubated at room temperature for 30 minutes in the dark, after which time 100 *μ*L of stop solution was added and the absorbance read at 450 nm.

### 2.4. Cell Migration Assay

Either control or knockdown human pancreatic cancer cells were grown to confluence in 10 cm tissue culture plates and then pretreated with *TNFα* with or without GW501516 for 24 h, as above. Cell migration assays were performed using the CytoSelect 96-well cell invasion assay (basement membrane, fluorometric format) according to the manufacturer's instructions. Briefly, the basement membrane was allowed to reach room temperature for 30 minutes and rehydrated using warm, serum-free DMEM. Human pancreatic cancer cells were then seeded into each well at a density of 2 × 10^6^ cells/mL in serum-free media. Normal cell media (DMEM containing 10% FBS, along with *TNFα* with or without GW501516) was added to the feeder tray, and the entire apparatus was placed in an incubator at 37°C containing 5% CO_2_ for 24 h. CyQuant GR dye/lysis buffer solution was added to the invading cells following completion of the assay, and the resulting mixture was incubated at room temperature for 20 minutes. Invading cells were quantified by reading the fluorescence at 480 nm/520 nm. All measurements were performed in triplicate. 

### 2.5. Lentiviral shRNA Infection

HEK-293 cells were grown to confluency in 10 cm tissue culture plates under the conditions described above. The cells were then transiently transfected with 4.6 *μ*g of either nontargeting shRNA or shRNAs targeted against human *PPARβ*/*δ, BCL-6, or MMP-9*, as well as 2.4 *μ*g each of pPACKH1 packaging plasmids, using Lipofectamine 2000. Cells were transfected for 6 h and allowed to recover overnight in normal media. Fresh media was added the following morning, and pseudoviral supernatant was generated for 72 h. Supernatant was then harvested and passed through a 0.4 *μ*m filter under sterile conditions. Polybrene (Millipore, Billerica, MA) was then added to a final concentration of 5 *μ*g per ml, and the pseudoviral supernatant was then added directly to target cells for 6 h. Infected cells were allowed to recover overnight following the addition of 6 mL complete media, and knockdown of target genes was assessed by RT-PCR 48 h postinfection. 

### 2.6. Statistical Analysis

Quantitative data are presented as mean ± SEM. ANOVA with *P*-value <0.05 was used to determine whether differences among variables were significant. Normality was checked using Anderson-Darling test and the general linear model, followed by the Tukey post hoc test to analyze differences between treatments. All data analyses were performed by MiniTAB Ver.14 (MiniTAB, State College, PA) or JMP (SAS Institute, Cary, NC), and data were plotted by Prism 5.01 (GraphPad Software, San Diego, CA).

## 3. Results

### 3.1. Tissue Samples from Human Pancreatic Ductal Carcinomas Show Significantly Increased Levels of *MMP*-*9* mRNA

**Figure 1:**
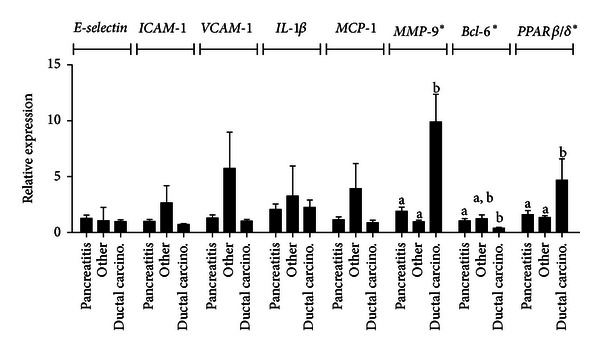
Relative mRNA expression in human pancreatic tissues at varying stages of carcinogenesis from chronic pancreatitis to pancreatic cancer. Total mRNA was isolated using standard Tri-Reagent protocol and reverse-transcribed. Gene expression was determined using qRT-PCR and expressed as fold induction after normalization to *β-actin*. *P < 0.05.

It is well known that the matrix metalloproteinases are key regulators of cell proliferation and migration in human pancreatic cancer cells [34] and that *MMP-9* protein is increased in the pancreatic juice from patients diagnosed with pancreatic ductal adenocarcinomas [35]. Recently, *MMP-9* has been linked to *PPARβ*/*δ* and the transcriptional repressor *BCL-6*; in *PPARβ*/*δ* ^−/−^ macrophages, for example, there was lower *MMP-9* expression compared with wild-type cells [15]. Tissue samples from patients diagnosed with chronic pancreatitis or pancreatic cancer were obtained, and we set out to assess the differences in expression of several genes involved in inflammation and metastasis. Indeed, there was a 10-fold increase in *MMP-9* gene expression in ductal carcinomas compared with samples from patients diagnosed with chronic pancreatitis (Figure 1). Interestingly, *PPARβ*/*δ* expression was also elevated while mRNA expression of the transcriptional repressor *BCL-6 *was almost 3-fold lower in tumor samples compared with those from chronic pancreatitis patients. Despite the low expression of *BCL-6 *in ductal carcinomas, the relative expression of two *BCL-6* target genes, *VCAM-1 *[36] and *MCP-1*, was not significantly elevated in tumor samples. A *PPARβ*/*δ* target gene, *ADRP*, was also not different between tumor, pancreatitis, and other pancreatic tissue samples (data not shown).

### 3.2. Regulation of *MMP-9* Expression by *PPARβ*/*δ* and *BCL6* in Pancreatic Cancer Cells

**Figure 2:**
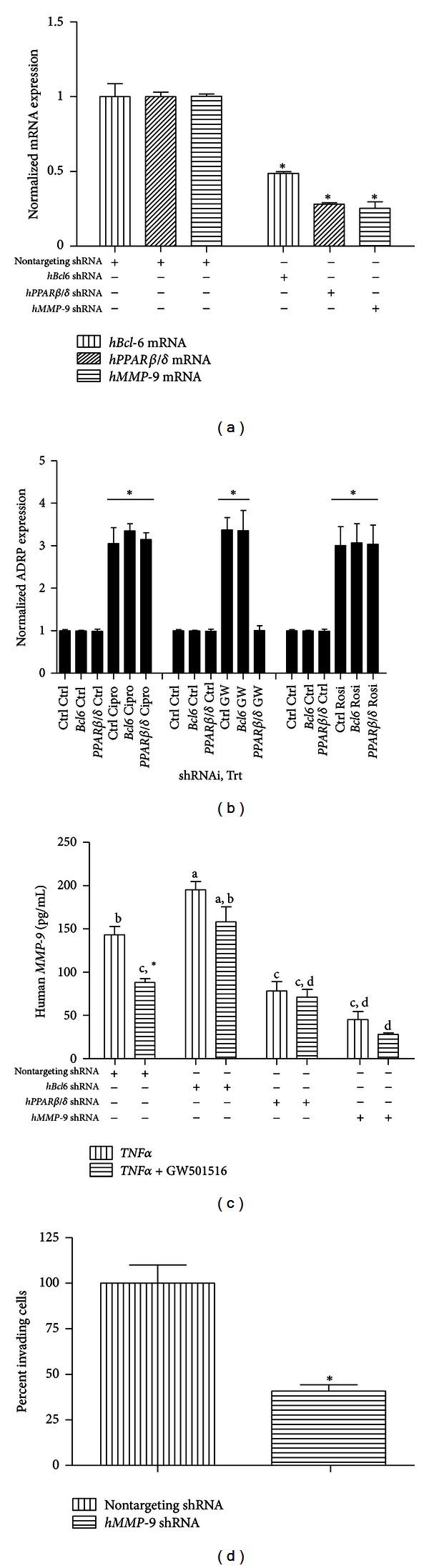
Effect of *PPARβ*/*δ* activation on *MMP-9* expression. (a) Miapaca-2 cells were transiently infected with nontargeting control, *hBCL-6,* *h* *PPARβ*/*δ*, or *hMMP9* lentiviral shRNAs for 48 hours. Total mRNA was isolated and gene-specific knockdown was assessed using qRT-PCR. *P < 0.05. (b) Miapaca-2 cells contain functional *PPAR*s. Miapaca-2 cells were transiently infected with the indicated shRNAs and treated with the indicated *PPAR* isoform-specific agonists. Induction of the *PPAR*-target gene *ADRP* was determined using qRT-PCR. *P < 0.05. (c) Miapaca-2 cells were stimulated with human *TNFα* with or without GW501516 for 24 hours following transient infection with the indicated shRNAs. Human *MMP-9* protein expression was quantified using *MMP-9*-specific ELISA (Invitrogen). *P < 0.05. (d) Miapaca-2 cells with reduced *MMP-9* expression are less invasive than control Miapaca-2 cells. Following infection with human *MMP-9*-targeting shRNA, Miapaca-2 cells were seeded in 96-well invasion plates (Cell Biolabs, Inc.) and allowed to invade the basement membrane overnight. Relative cell invasion was quantified using the CytoSelect 96-well cell invasion assay with fluorometric readings at 480 nm/520 nm. *P < 0.05.

*PP* *AR* *β*/*δ* activation negatively influences *MMP-9 *gene expression in *IL-1*β*-*stimulated vascular smooth muscle cells [23]. To study the mechanism of this response and to determine its applicability to another cell type, Miapaca-2 cells were transiently infected with nontargeting control, *hBCL-6*, *h* *PPARβ*/*δ*, or *hMMP9* lentiviral shRNAs (Figure 2(a)). Cells transiently infected with nontargeting control shRNA showed no alterations in either *BCL-6,* *PPARβ*/*δ*, or *MMP9* mRNA expression. Miapaca cells infected with an shRNA targeted against *BCL-6 *showed approximately 50% reduction in *BCL-6 *mRNA levels, and cells infected with an shRNA targeting *PPARβ*/*δ* or *MMP9* showed approximately 70% reduction in corresponding mRNA levels (Figure 2(a)). To determine if gene expression is altered after lentiviral-mediated *BCL-6 *or *PPARβ*/*δ* repression, the *PPAR* target gene *ADRP* was examined upon treatment with three isoform-specific *PPAR* agonists (ciprofibrate, *PPARα*; GW501516, *PPARβ*/*δ*; rosiglitazone, *PPARγ*). Control cells and those transiently expressing the indicated shRNAs were treated with either 20 *μ*M ciprofibrate, 500 nM GW501516, or 10 *μ*M rosiglitazone. Miapaca-2 cells contain functional *PPAR*s as indicated by the ligand-induced expression of *ADRP*, with each treatment resulting in a three-fold increase in transcript levels (Figure 2(b)). Cells expressing *PPARβ*/*δ-*specific shRNA did not induce expression of *ADRP* in response to GW501516 at a concentration that activates only *PPARβ*/*δ* [37], while cells expressing *BCL-6*-targeting shRNA retained inducible expression of *ADRP* by all three isoform-specific ligands. Following *BCL-6*, *PPARβ*/*δ* or *MMP-9* knockdown, cells were treated with 1 ng/mL *TNFα* with or without 500 nM GW501516 for 24 hours, and *MMP-9* protein levels were assessed by ELISA. *MMP-9* protein levels were significantly elevated in *BCL-6* knockdown Miapaca-2 cells following *TNFα* challenge compared with control cells, while *PPARβ*/*δ* knockdown Miapaca-2 cells showed a significant reduction in *TNFα*-induced *MMP-9* protein levels (Figure 2(c)), consistent with previous reports in *PPARβ*/*δ* ^−/−^ macrophages. While GW501516 cotreatment significantly suppressed *TNFα*-induced *MMP-9* protein levels in control (nontargeting) Miapaca-2 cells, this effect was not observed in either of the *BCL-6* or *PPARβ*/*δ* knockdown cells. Not unexpectedly, lentiviral shRNA targeted against *MMP-9* significantly reduced both mRNA and protein expression in Miapaca-2 cells, and GW501516 activation of *PPARβ*/*δ* did not further reduce *MMP-9* protein levels in these cells.

### 3.3. *MMP-9* Knockdown Reduces Miapaca-2 Cell Invasion

Because *MMP-9* is a key regulator of human pancreatic cancer cell invasion and metastasis, we further examined the effect of lentiviral shRNA-mediated *MMP-9* knockdown on the basal ability of Miapaca-2 cells to invade a basement membrane. Miapaca-2 cells treated with non-targeting control or *MMP-9*-targeting lentiviral shRNAs were seeded into a 96-well invasion plate and allowed to migrate across a membrane for 24 hours. Using the migration assay described, we found that *MMP-9* knock-down significantly reduced the basal migration of Miapaca-2 cells (Figure 2(d)).

### 3.4. *PPARβ*/*δ* Activation Decreases *TNF*α**-Induced Expression of Proinflammatory and Cell Adhesion Genes in Human Pancreatic Cancer Cells

**Figure 3:**
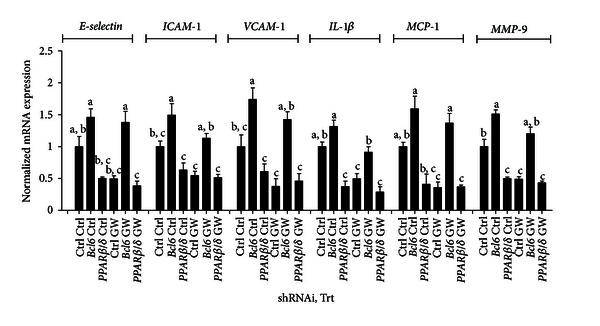
Effects of *PPARβ*/*δ* and *BCL-6* knockdown on Miapaca-2 gene expression. Miapaca-2 cells were transiently infected with nontargeting control, *hBCL-6* or *h* *PPARβ*/*δ-*specific shRNAs and then stimulated with human *TNFα* with or without GW501516 for 24 hours. Total mRNA was isolated and gene expression was determined using qRT-PCR. Data is normalized to *β*-actin and indicated as fold change.

*PP* *AR* *β*/*δ* associates with the transcriptional repressor *BCL-6* which, upon *PPARβ*/*δ* activation, is released and decreases expression of target genes. To determine if the *PPARβ*/*δ*/*BCL-6* pathway is active in human pancreatic cancer cells, we used shRNA knock-down of *PPARβ*/*δ* and *BCL-6* in conjunction with *PPARβ*/*δ-*specific activation by GW501516 to analyze the gene expression changes. Miapaca-2 cells transiently expressing non-targeting control shRNA, or shRNAs targeted against *PPARβ*/*δ* or *BCL-6*, were stimulated with 1 ng/mL *TNFα* with or without GW501516 for 24 hours. In control Miapaca-2 cells, *TNFα* stimulation induced the robust expression of the cell adhesion molecules *E-selectin*, *ICAM-1* and *VCAM-1*, the proinflammatory genes *IL-1*β**and *MCP-1,* and the promigratory gene *MMP-9*, while cotreatment with 500 nM GW501516 significantly suppressed their expression at the mRNA level (Figure 3). Treatment of Miapaca-2 cells with a *BCL-6*-targeting shRNA attenuated the GW501516 inhibitory effect on the genes tested, indicating a role for *BCL-6* in GW501516-mediated repression. Consistent with the findings of Lee et al. in *PPARβ*/*δ* ^−/−^ RAW264.7 macrophage cells, *PPARβ*/*δ* knock-down Miapaca-2 cells displayed significantly lower levels of these genes when challenged with *TNFα* alone, and *PPARβ*/*δ* activation with GW501516 had no further significant repressive effect. Of note is the fact that although *BCL-6* repression resulted in increased *MMP-9* protein levels in media, it did not concordantly increase the mRNA expression of this gene.

### 3.5. *PPARβ*/*δ* Activation Inhibits Human Pancreatic Cancer Cell Migration

**Figure 4:**
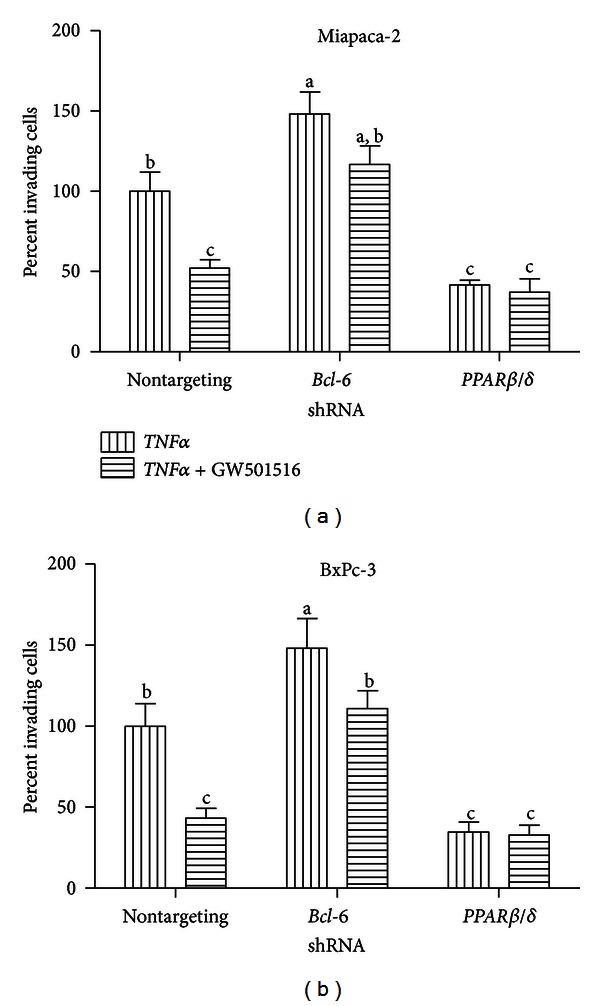
GW501516 treatment reduces *TNFα*-stimulated Miapaca-2 and BxPc-3 cell invasion through a basement membrane. Human pancreatic cancer cells were transiently infected with the indicated shRNAs and stimulated with human *TNFα* with or without GW501516. Cells were allowed to invade a basement membrane overnight, and relative invasion was quantified using the CytoSelect 96-well cell invasion assay with fluorometric readings at 480 nm/520 nm. GW501516 treatment inhibits the invasion of the *COX-2* negative pancreatic cancer cell line Miapaca-2 (a) and the *COX-2* positive pancreatic cancer cell line BxPc-3 (b) through a basement membrane.

To examine if *PPARβ*/*δ* activation by GW501516 and subsequent repression of pro-inflammatory and pro-migratory genes via *BCL-6* influenced their ability to invade a basement membrane, Miapaca-2 (*COX-2* negative, Figure 4(a)) and BxPc-3 (*COX-2* positive, Figure 4(b)) were treated with non-, *PPARβ*/*δ-* or *BCL-6*-targeting shRNA, and the effects of GW501516 on cell migration were examined. In control cells, GW501516 treatment negatively influenced the ability of either Miapaca-2 or BxPc-3 cells to migrate across a membrane (50% reduction). Lentiviral-mediated knock-down of *BCL-6*, however, increased cell migration in both cell lines compared with control cells. GW501516 treatment of *BCL-6 *repressed cells did not have an effect on Miapaca (Figure 4(a)) but did have an effect on comparable BxPc-3 cells (Figure 4(b)). Interestingly, Miapaca-2 and BxPc-3 cells transiently expressing an shRNA targeted against *PPARβ*/*δ* showed significantly reduced cell migration compared with control cells with or without GW501516 treatment. These results suggested that the transcriptional repressor *BCL-6 *mediates the antimigratory actions of GW501516 in human pancreatic cancer cells but does so in a *PPARβ*/*δ*-dependent manner.

## 4. Discussion

The *PPAR* nuclear receptors are regulators of inflammation and proliferation in human pancreatic cells [16, 38, 39]. Although several studies implicate *PPARγ* activation in inhibition of pancreatic cancer cell growth, little is known about the role of *PPARβ*/*δ*, save for its role in suppressing inflammation via *BCL-6* [16]. Generally, the role of *PPARβ*/*δ* in cancer cell growth and tumorigenesis remains controversial. In colorectal cancer cells, nonsteroidal anti-inflammatory drugs inhibit tumorigenesis through inhibition of *PPARβ*/*δ* [27], and *PPARβ*/*δ* promotes intestinal carcinogenesis [40]. Studies in the *PPARβ*/*δ* null mouse, however, show that activation of *PPARβ*/*δ* induces terminal differentiation [41], and *PPARβ*/*δ-*specific ligands inhibit the growth of keratinocytes *in vivo* [42, 43] and *in vitro* [32]. Furthermore, *PPARβ*/*δ* activation is linked to inhibition of *IL-1*β*-*stimulated proliferation and migration of vascular smooth muscle cells [23] via regulation of *IL-1Ra* and *TGF*-*β* and negative regulation of *MMP-9*. Our results show that *PPARβ*/*δ* activation by GW501516 suppresses expression of *MMP-9* in human pancreatic cancer cells via *BCL-6*, with further inhibition on the ability of two cell lines, Miapaca-2 and BxPc-3, to invade a basement membrane. 

Consistent with previous work [35], analysis of the expression levels of several genes in both ductal carcinomas and chronic pancreatitis showed elevated levels of the matrix-remodeling gene *MMP-9*. Several studies link increased *MMP-9 *levels to increased invasiveness and metastasis [44, 45]. *MMP-9 *is a critical player in the early stages of tumor invasion by degrading basement membrane type IV collagen [46], considered to be a crucial step in tumor cell invasion [47]. *MMP-9 *also participates in the degradation of the various components of the ECM [48]. Inhibition of *MMP* activity by orally bioavailable matrix metalloproteinase inhibitors has shown promise in decreasing tumor metastasis in clinical trials [46]. In the cell lines BxPc-3 and Miapaca-2, treatment with the neurotransmitter norepinephrine increased cell invasiveness via augmented *MMP-2, MMP-9*, and *VEGF* [49], while treatment with the *β*-blocker propranolol inhibited these effects. Clearly the regulation of *MMP* activity is important in controlling, and possibly treating, pancreatic cancer. The association between *MMP-9*,*PPARβ*/*δ*, and *BCL-6* was established by Lee et al. [15], demonstrating that *MMP-9* expression in *PPARβ*/*δ* ^−/−^ macrophages is repressed compared to wild type. Activation of the receptor significantly decreased the expression of pro-inflammatory markers, suggesting that *BCL-6* released from the *PPARβ*/*δ* complex plays a role in the regulation of *MMP-9*. A similar result was obtained in VSMCs [23]. 

In the present studies, *PPARβ*/*δ* mRNA were increased in ductal carcinomas, while *BCL-6* expression was decreased. In colorectal cancer tissue samples, *PPARβ*/*δ* expression increased during multistage carcinogenesis and was tightly associated with a highly malignant morphology [50]. It is possible that *PPARβ*/*δ* plays a role in human pancreatic cells, but whether *PPARβ*/*δ* contributes to pancreatic cancer cell metastasis or if its overexpression is the result of some altered signaling pathway remains unclear. Since the regulatory region of the *PPARβ*/*δ* contains several AP-1 response elements and is controlled by a variety of inflammatory signals [51], the increased expression of this nuclear receptor may be indicative of stress response and not causally related to the tumor phenotype. Increased expression of *PPARβ*/*δ* in the inactivated state may sequester *BCL-6*, increasing the expression of genes normally controlled by this transcriptional repressor. 

Of particular note is the observation that the relative expression of *BCL-6*, a proto-oncogene known to suppress genes involved in cell cycle progression, particularly cyclin D1 [52], and inflammation [53], was lower in ductal carcinomas compared with pancreatitis. *BCL-6* is mutated in several disorders [54–56] and is implicated in cell line immortalization and transformation by overriding cellular senescence downstream of *p53* [57]. Interestingly, DNA-chip hybridization assays identified both *BCL-6* and *BCL-10* as novel candidate genes in pancreatic cancer that were overexpressed in pancreatic cancer cell lines and primary tumor samples [58]. Contrary to ductal cells, *BCL-6* is absent in pancreatic beta cells, potentially explaining the lack of anti-inflammatory *PPARβ*/*δ* signaling in this cell type [16]. Our results suggest that the *BCL-6* pathway is disrupted as inflamed tissue transforms into tumor, with the possibility that loss of *BCL-6* expression leads to increased cancer invasion via increased *MMP-9* expression. 

Activation of *PPARβ*/*δ* decreased the expression of *MMP-9* at the protein level, while knockdown of *BCL-6* increased *MMP-9* protein production. Activation of *PPARβ*/*δ* in *BCL-6* knock-down cells showed no significant effect on reducing *MMP-9* protein, suggesting a key role for *BCL-6* in the regulation of *MMP-9*. Knocking down *PPARβ*/*δ* in human pancreatic cancer cells reduced *MMP-9* protein levels to those comparable to GW501516-treated control cells despite *PPARβ*/*δ* activation. It is our hypothesis that *MMP-9* is an indirect *PPARβ*/*δ* target gene and that *PPARβ*/*δ* activation releases *BCL-6* which may then relocate to the *MMP-9* promoter. Further studies, such as chromatin immunoprecipitation assays, for example, are required to substantiate this point. Our results, however, are in agreement with previous work indicating that low levels of *PPARβ*/*δ* result in decreased *MMP-9* expression [15], and we believe that, in the absence of *PPARβ*/*δ, BCL-6* is available to repress target genes, either through direct repression on target gene promoters as in the case of *VCAM-1* and *E-selectin*, two genes lacking PPREs, [36] or through interactions with other cell signaling mediators, such as *NF*-*κB* [59]. GW0742 has been found to inhibit inflammation by *T* *NF*-*α* while not preventing *T* *NF*-*α*-induced degradation of *I* *κB* *α* and the translocation of *NF* *κB*. No decrease in DNA binding activity indicates *PPARβ*/*δ* must interfere via a corepressors mechanism at the chromatin level [36].

The relationship between *MMP-9* and metastasis in pancreatic cancer is well documented. Treatment of Miapaca-2 cells with *MMP-9* shRNA reduced protein levels by approximately 50% regardless of GW501516 treatment, with corresponding inhibition on the ability of the cell line to invade a basement membrane. Miapaca-2 cells are considered a highly metastatic cell line [60], and our results support the idea that regulating *MMP-9* expression may effectively control human pancreatic cancer cell invasion and metastasis.

*PP* *AR* *β*/*δ* activation is associated with reduced inflammatory and adhesion cell markers. Our results in Miapaca-2 cells show that the *PPARβ*/*δ* BCL-6  anti-inflammatory pathway is active and represses *E-selectin, ICAM-1, VCAM-1, IL-1*β*, MCP-1, and MMP-9*. Repression of *VCAM-1, E-selectin,* [36], and *MCP-1* [15] in particular is dependent upon dissociation of *BCL-6* from *PPARβ*/*δ* and subsequent relocation to the corresponding promoters. Our results demonstrate that the proadhesion molecules *E-selectin, ICAM-1, and VCAM-1*, the pro-inflammatory *IL-1*β** and *MCP-1,* and the prometastasis gene *MMP-9* are *BCL-6*-regulated genes in human pancreatic cancer cells. Treatment with *BCL-6* shRNA attenuated the GW501516-mediated inhibition of *TNFα*-induced expression of these molecules, while treatment with *PPARβ*/*δ* shRNA reduced their expression at the mRNA level regardless of GW501516 treatment. *E-selectin*, *ICAM-1,* and *VCAM-1* are important in pancreatic cancer, where they participate in the detachment of cells from the primary tumor and contribute to cancer spread [61], and their overexpression in pancreatic adenocarcinomas is associated with a stimulation in tumor growth, increased metastatic ability, and potentially shorter postoperative survival following tumor resection [62]. *IL-1*β** induces *MMP-9* expression in other cell lines [63–65] and enhances the invasiveness of human pancreatic cancer cells [66]. Monocyte chemotactic protein-1 is produced by pancreatic cancer cells in response to *TNFα* challenge and may contribute to the accumulation of tumor-associated macrophages [67] which influence key events in the tumor invasion process [68]. Taken together, these results suggest that *PPARβ*/*δ* activation and subsequent suppression of proadhesion and pro-migratory genes via *BCL-6* might prove useful in the control of pancreatic cancer. 

GW501516 treatment also inhibited the *TNFα*-promoted invasion of a basement membrane by the pancreatic ductal cell lines Miapaca-2 and BxPc-3. Pancreatic cancer cells transiently expressing *BCL-6* shRNA were significantly more invasive, while GW501516 treatment attenuated their invasive potential close to control levels. Conversely, cells transiently expressing *PPARβ*/*δ* shRNA were significantly less invasive than control cells, and GW501516 treatment showed no significant effect in further reducing invasion. We hypothesize that cells expressing lower levels of the transcriptional repressor *BCL-6* are more invasive owing to a lack of control over pro-migratory gene regulation and increased protein levels of *MMP-9*, while knocking down *PPARβ*/*δ* via shRNA methods allow for a greater population of unassociated *BCL-6* which is available to repress pro-migratory genes resulting in lower invasion. Although we present here a fairly simplified mechanism, it is possible that more complex signaling cascade is taking place resulting in the inhibition of cell invasion. In VSMCs, repression of *MMP-9* activity was effected in a *TGF*-*β-*dependent manner following *PPARβ*/*δ* activation [23], and indeed, *TGF*-*β* suppresses *MMP-9* expression in monocytes through a prostaglandin E2- and cAMP-dependent mechanism [69]. *TGF*-*β* is a *PPARβ*/*δ* target gene in VSMCs [70], and it is possible that the PPAR*β*/*δ* *-TGF-*β** pathway could in fact be active in human pancreatic cancer cells. Our results suggest, however, that *BCL-6* and *PPARβ*/*δ* play critical roles in suppressing pro-migratory gene expression at the mRNA level and in ultimately controlling human pancreatic cancer cell invasion.

Our observations demonstrate that activation of *PPARβ*/*δ* by a specific agonist reduces the *TNFα*-induced mRNA levels of genes known to be involved in the regulation of human pancreatic cancer cell invasion and metastasis, and this negative regulation is also manifested at the protein level. Furthermore, we show that *PPARβ*/*δ* activation reduces Miapaca-2 and BxPc-3 invasion through a basement membrane, and that the transcriptional repressor *BCL-6* plays a critical role in the pathway(s) regulating human pancreatic cancer cell invasion. This is not the first time *PPAR* activators have been shown to negatively influence pancreatic cancer cell invasion, and perhaps further *in vivo* studies using these mouse transplantable cell lines could provide more useful insight into the potential therapeutic uses of *PPARβ*/*δ* activators in the control and regulation of pancreatic cancer.

## Supplementary Material

List of Real-time PCR primers used in this study.
