# Supplementary material for: Role of Peroxisome Proliferator-Activated Receptor β/δ and B-Cell Lymphoma-6 in Regulation of Genes Involved in Metastasis and Migration in Pancreatic Cancer Cells
Source: PPAR Res. 2013 May 2;2013:121956. doi: 10.1155/2013/121956 (PMC3659435; doi:10.1155/2013/121956)
Supplement: Supplementary file 1 — List of Real-time PCR primers used in this study. [file 121956.f1.docx]

**Table 1**. List of Real-time PCR primers used in this study.

| Gene | Forward primer | Reverse primer |
| --- | --- | --- |
| *ADRP*  *β-Actin* | TGACTGGCAGTGTGGAGAAGA  AACAAGAGGCCACACAAATAGG | TGTTAATGCTGCCACTGACCA  CAGATGTACAGGAATAGCCTCCG |
| *E-selectin* | TCCTATTCCAGCCTGCAATGT | AACCCATTGGCTGGATTTGTC |
| *ICAM-1* | ACTCAGCGGTCATGTCTGGAC | GGCATAGCTTGGGCATATTCC |
| *VCAM-1* | AGTGGTGGCCTCGTGAATG | CACGCTAGGAACCTTGCAGC |
| *IL-1β* | TCCTTAGTCCTCGGCCAAGAC | GTGCCATGGTTTCTTGTGACC |
| *MCP-1* | GGACGCATTTCCCCAGTACA | CCGAGAACGAGATGTGGACA |
| *MMP-9* | AGCGGTCCTGGCAGAAATAG | ACGCACGACGTCTTCCAGTAC |
| *BCL-6* | GCTCACGGCTCACAACAATG | TCCGGAGTCGAGACATCTTGA |
| *PPARβ/δ* | AGGCCATTCACCAACTGCTT | ATTGTGGCAGGCAGAGAAGG |
